# Notch signalling defines dorsal root ganglia neuroglial fate choice during early neural crest cell migration

**DOI:** 10.1186/s12868-019-0501-0

**Published:** 2019-04-29

**Authors:** Sophie Wiszniak, Quenten Schwarz

**Affiliations:** Centre for Cancer Biology, University of South Australia and SA Pathology, North Terrace, Adelaide, SA 5001 Australia

**Keywords:** Notch signalling, Mib1, Neural crest, Fate restriction, Dorsal root ganglia

## Abstract

**Background:**

The dorsal root ganglia (DRG) are a critical component of the peripheral nervous system, and function to relay somatosensory information from the body’s periphery to sensory perception centres within the brain. The DRG are primarily comprised of two cell types, sensory neurons and glia, both of which are neural crest-derived. Notch signalling is known to play an essential role in defining the neuronal or glial fate of bipotent neural crest progenitors that migrate from the dorsal ridge of the neural tube to the sites of the DRG. However, the involvement of Notch ligands in this process and the timing at which neuronal versus glial fate is acquired has remained uncertain.

**Results:**

We have used tissue specific knockout of the E3 ubiquitin ligase mindbomb1 (Mib1) to remove the function of all Notch ligands in neural crest cells. *Wnt1*-*Cre*; *Mib1*^*fl*/*fl*^ mice exhibit severe DRG defects, including a reduction in glial cells, and neuronal cell death later in development. By comparing formation of sensory neurons and glia with the expression and activation of Notch signalling in these mice, we define a critical period during embryonic development in which early migrating neural crest cells become biased toward neuronal and glial phenotypes.

**Conclusions:**

We demonstrate active Notch signalling between neural crest progenitors as soon as trunk neural crest cells delaminate from the neural tube and during their early migration toward the site of the DRG. This data brings into question the timing of neuroglial fate specification in the DRG and suggest that it may occur much earlier than originally considered.

**Electronic supplementary material:**

The online version of this article (10.1186/s12868-019-0501-0) contains supplementary material, which is available to authorized users.

## Background

Peripheral sensory neurons and their supporting glia condense into specialised dorsal root ganglia (DRG) that transmit somatosensory information from the body’s periphery to sensory perception centres within the central nervous system. A diverse array of neuronal sub-types underlies the ability of the DRG to transduce different sensations including pain, proprioception, temperature and touch. Satellite glia sit in close association with neuronal cell bodies of the sensory ganglia to modulate their microenvironment [1]. Both cell types comprising the DRG arise from a sub-population of bipotent trunk neural crest cell progenitors that are biased towards the sensory lineage [2]. This population of trunk neural crest cells migrates ventrally from the dorsal neural tube, arrests within the somites, and is then thought to gain neuronal or glial identity as they coalesce into the DRG [3, 4].

Notch signalling is known to play an important role in neuroglial fate choice determination [5, 6]. The Notch signalling pathway relies on direct cell–cell interactions mediated by Notch receptors on the signal-receiving cell and ligands such as Delta and Jagged on the signal-sending cell. By a process known as lateral inhibition, Notch signalling enables a population of homogeneous progenitors to become specified into different cell types, namely neurons and glia in the developing nervous system. In the DRG, cells expressing Delta-like 1 (DLL1) adopt a neuronal fate, which then signal to adjacent cells via their Notch receptors to inhibit neuronal differentiation. This in turn promotes glial differentiation of cells in which neuronal differentiation has been inhibited [7, 8].

The study of Notch signalling in DRG development has been complicated by the presence of multiple Notch receptors and ligands, all of which have the potential to act in a functionally redundant manner. This has been overcome to some degree by the study of recombination signal binding protein for immunoglobulin kappa J region (*Rbpj*) conditional knockout mice. RBPJ is a transcription factor which interacts with the intracellular domain of all Notch receptors and is essential to mediate downstream transcriptional effects upon Notch pathway stimulation. Therefore, knockout of *Rbpj* is expected to abolish all Notch signalling. Removal of *Rbpj* specifically in neural crest cells leads to profound DRG defects, including a significant reduction in glial cells [9, 10], which is consistent with a role for Notch signalling in promoting glial cell development. However, these studies did not define the timing of Notch activation during DRG development and gliogenesis, as well as the roles for Notch ligands in this process.

While several Notch ligands have been ubiquitously removed during mouse development (e.g. *Dll1*-*null*) their specific effects on DRG formation have been difficult to study given the broad roles Notch ligands play in many organ systems during embryonic development, and the severity of knockout phenotypes [11]. Also, the removal of individual Notch ligands may be phenotypically misleading if multiple ligands are able to function in a redundant fashion. To overcome these difficulties, we addressed the function of Notch ligands during neural crest and DRG development by removing *Mind bomb 1* (*Mib1*) conditionally in neural crest-derived tissue. Mib1 is an E3 ubiquitin ligase that is required in signal-sending cells to target the Notch ligands for ubiquitination. This ubiquitination is required for the internalisation of Notch ligands upon signalling to Notch receptors on adjacent cells, and this internalisation is essential for the correct cleavage and translocation of the Notch intracellular domain to the nucleus in the signal-receiving cell to enable active Notch signalling [12]. Therefore, removal of *Mib1* in neural crest cells is expected to abolish activity of all Notch ligands.

Consistent with the known role for Notch signalling in DRG development, *Wnt1*-*Cre*; *Mib1*^*fl*/*fl*^ embryos exhibit severe DRG hypoplasia, with a dramatic reduction in glial cells in the DRG. Interestingly, the loss of glial cells was preceded by reduced SRY-related HMG-box 10 (Sox10) expression in a subset of early migrating neural crest cells. Sox10 is a marker of pre- and migratory neural crest cells, which is lost as neural crest cells differentiate into neurons, however is maintained in neural crest progenitors that differentiate into glia, and thus is also used as a marker of mature glial cells [13, 14]. This early loss of Sox10 presents the possibility that a subset of neural crest cells are specified to become glia at the earliest stages of their migration, before neurons differentiate. In the absence of *Mib1*, DLL1 protein accumulates at the cell membrane, and enables visualisation of neuronally biased signal-sending cells attempting to undergo active Notch signalling. In *Wnt1*-*Cre*; *Mib1*^*fl*/*fl*^ embryos, aberrant DLL1 accumulation was evident as early as E9.25 immediately after neural crest cells had delaminated from the neural tube, and this was accompanied by a loss of Notch1 intracellular domain (N1ICD) in the nucleus of migrating neural crest cells at this stage. This is the first study to demonstrate active Notch signalling between neural crest progenitors at this early stage of trunk neural crest migration, and suggests that the signalling events controlling neuroglial fate specification in the DRG occur much earlier than originally considered.

## Results

### Loss of Mib1 in neural crest cells causes severe dorsal root ganglia hypoplasia

**Fig. 1:**
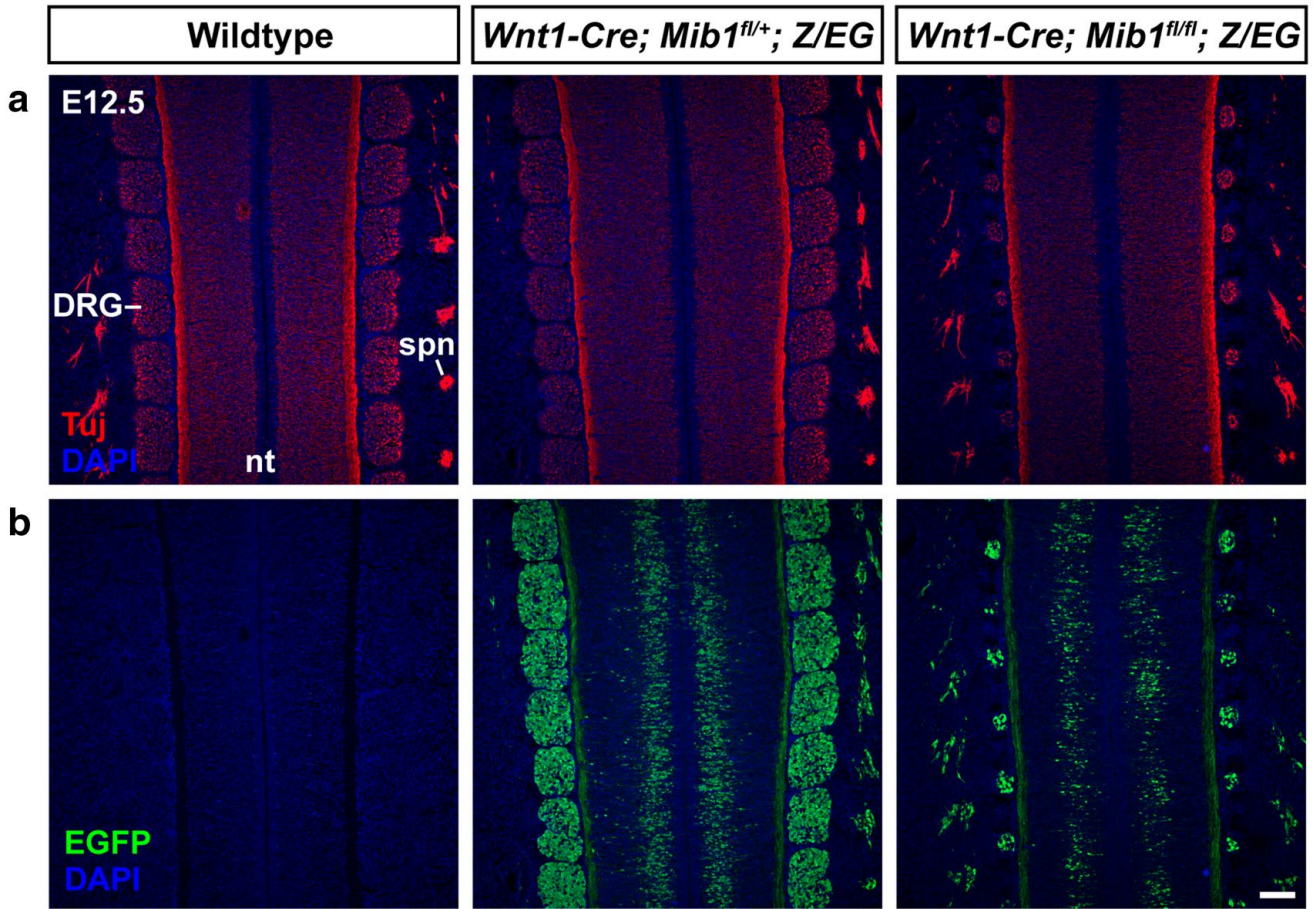
Loss of Mib1 in neural crest cells causes severe DRG hypoplasia. Longitudinal sections over the forelimb region of E12.5 wildtype, *Wnt1*-*Cre*; *Mib1*^*fl*/+^; *Z*/*EG*, and *Wnt1*-*Cre*; *Mib1*^*fl*/*fl*^; *Z*/*EG* embryos were co-immunostained for Tuj1 (**a**) and EGFP (**b**) n = 3/genotype. Blue, DAPI. Colour channels are separated for clarity. nt, neural tube; spn, spinal nerve. Scale bar = 100 μm

At E12.5, the dorsal root ganglia (DRG) appear as uniformly-shaped, segmented tissue structures, positioned bi-laterally adjacent to the neural tube. All neurons and glia that comprise the mature DRG are derived from neural crest cells [15]. Removal of Mib1 specifically in neural crest-derived tissue using a *Wnt1*-*Cre* driver and *Mib1*^*fl*^ allele revealed severe DRG hypoplasia (n = 3/3; Fig. 1). Longitudinal sections through the developing neural tube and DRG at E12.5 revealed that compared to control littermates (*Wildtype* (Cre-negative) and *Wnt1*-*Cre*; *Mib1*^*fl*/+^), *Wnt1*-*Cre*; *Mib1*^*fl*/*fl*^ embryos exhibited a dramatic reduction of DRG neuronal tissue, as labelled by immunostaining for the mature neuronal marker Tuj1 (Fig. 1a). Lineage tracing of neural crest-derived tissue was performed by examining expression of a *Z*/*EG* reporter gene, which permanently labels cells with EGFP upon expression of the *Wnt1*-*Cre* driver. Similar to Tuj1, EGFP immunostaining was dramatically reduced in the DRG of *Wnt1*-*Cre*; *Mib1*^*fl*/*fl*^ embryos (Fig. 1b), indicating DRG deficiency at E12.5 is caused by a loss of all neural crest-derived cellular components of the DRG.

### Loss of Mib1 results in reduced glial progenitors, premature neuronal differentiation and neuronal cell death in DRG

**Fig. 2:**
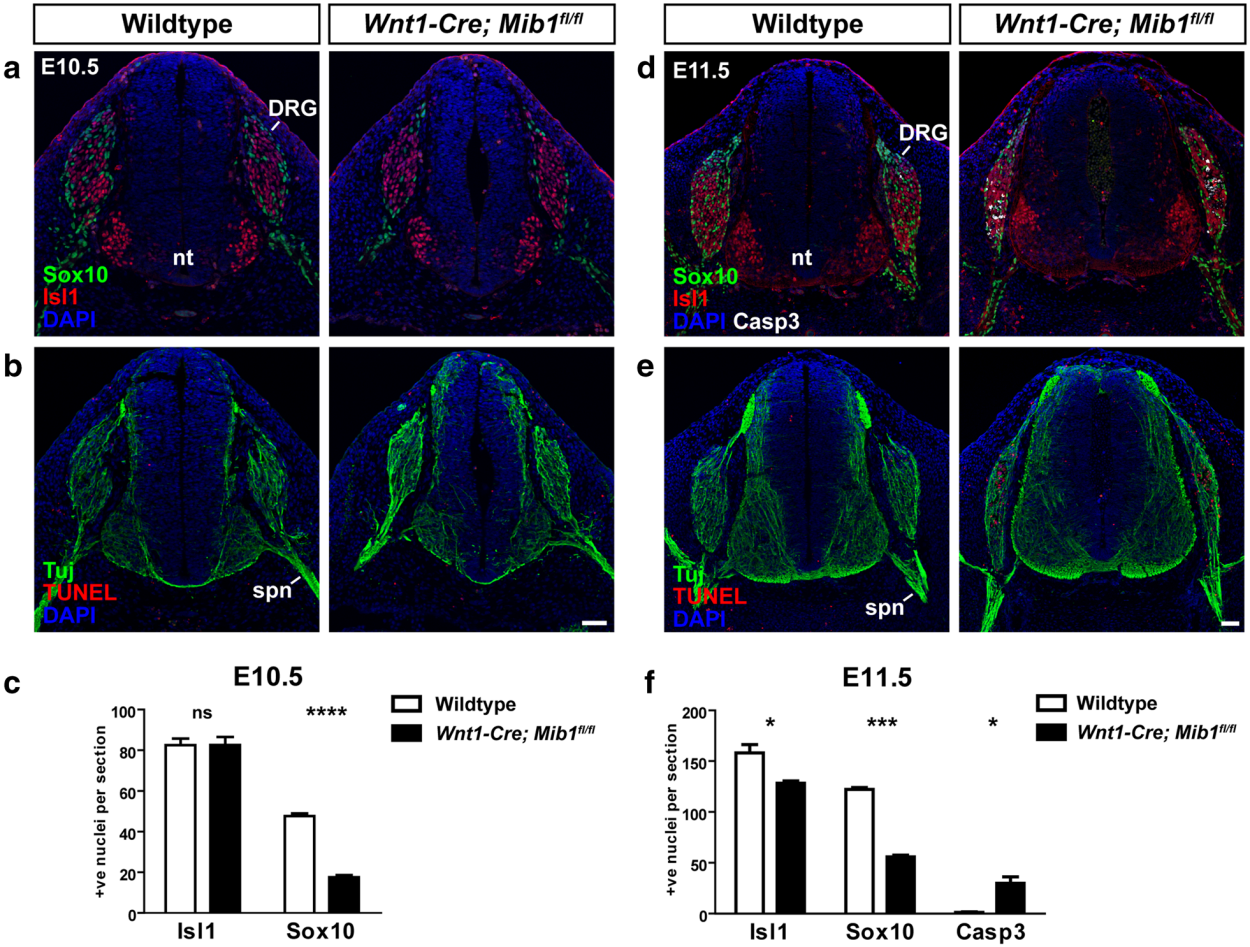
Loss of Mib1 causes a reduction in glial progenitors and DRG cell death. **a** Transverse sections over the forelimb region of wildtype and *Wnt1*-*Cre*; *Mib1*^*fl*/*fl*^ embryos at E10.5 co-immunostained for the glial marker Sox10 and the neuronal marker Isl1. **b** Serial section of A co-immunostained for the axonal marker Tuj1 and cell death marker TUNEL. Scale bar = 50 μm. **c** Quantitation of Isl1-positive and Sox10-positive cells per DRG at E10.5. Number represents mean of n = 3 embryos, with over 20 individual sections counted per embryo, *ns* not significant. ****p < 0.0001. **d** Transverse sections over the forelimb region of wildtype and *Wnt1*-*Cre*; *Mib1*^*fl*/*fl*^ embryos at E11.5 co-immunostained for Sox10, Isl1 and the cell death marker cleaved-Caspase 3 (Casp3). **e** Serial section of D co-immunostained for Tuj1 and TUNEL. Scale bar = 50 μm. **f** Quantitation of Isl1-positive, Sox10-positive and Casp3-positive cells per DRG at E11.5. Number represents mean of n = 3 embryos, with over 20 individual sections counted per embryo, *nt* neural tube, *spn* spinal nerve. *p < 0.05, ***p < 0.001

To investigate the developmental timing of DRG deficiency, DRG formation was examined at earlier stages of development. By E10.5, all neural crest cells that will contribute to the neuronal and glial cell subtypes of the DRG have delaminated from the neural tube, and reside as a mass of cells lateral to the spinal cord. At this stage of DRG development, neuronal and glial progenitors have begun the process of specification and differentiation as defined by the markers Isl1 for neurons and Sox10 for glial cells. Analysis of transverse sections through the DRG over the forelimb region of E10.5 wildtype and *Wnt1*-*Cre*; *Mib1*^*fl*/*fl*^ embryos revealed a dramatic reduction in Sox10-positive glial progenitors in *Wnt1*-*Cre*; *Mib1*^*fl*/*fl*^ embryos, without a change in the number of Isl1-positive neurons (n = 3/3; Fig. 2a, c). Immunostaining of serial sections with the neuronal marker Tuj1, further showed that neuronal differentiation proceeded normally in the DRG at E10.5 (Fig. 2b). No changes in cell death, assessed by Terminal deoxynucleotidyl transferase dUTP nick end labelling (TUNEL), were observed that may explain the loss of Sox10-positive glial progenitors (Fig. 2b).

By E11.5, the number of Sox10-positive glia and Isl1-postive neurons was significantly reduced in *Wnt1*-*Cre*; *Mib1*^*fl*/*fl*^ embryos compared to wildtype (n = 3/3; Fig. 2d, f). This was accompanied by a dramatic increase in cell death in the DRG, as assessed by cleaved-caspase 3 (Casp3) and TUNEL staining (Fig. 2d–f). Analysis of the mature glial marker Fatty acid binding protein 7 (FABP7) also showed that gliogenesis in the DRG was severely inhibited in *Wnt1*-*Cre*; *Mib1*^*fl*/*fl*^ embryos at both E11.5 and E13.5 (n = 3/3; Additional file 1: Fig. S1). Our data therefore suggest that the DRG hypoplasia observed in *Wnt1*-*Cre*; *Mib1*^*fl*/*fl*^ embryos from E11.5 occurs as a result of both glial deficiency and aberrant neuronal cell death. Whether this is due to a cell-autonomous role for Mib1 in neuronal cell survival, or a secondary effect due to loss of glial cells remains to be investigated.

**Fig. 3:**
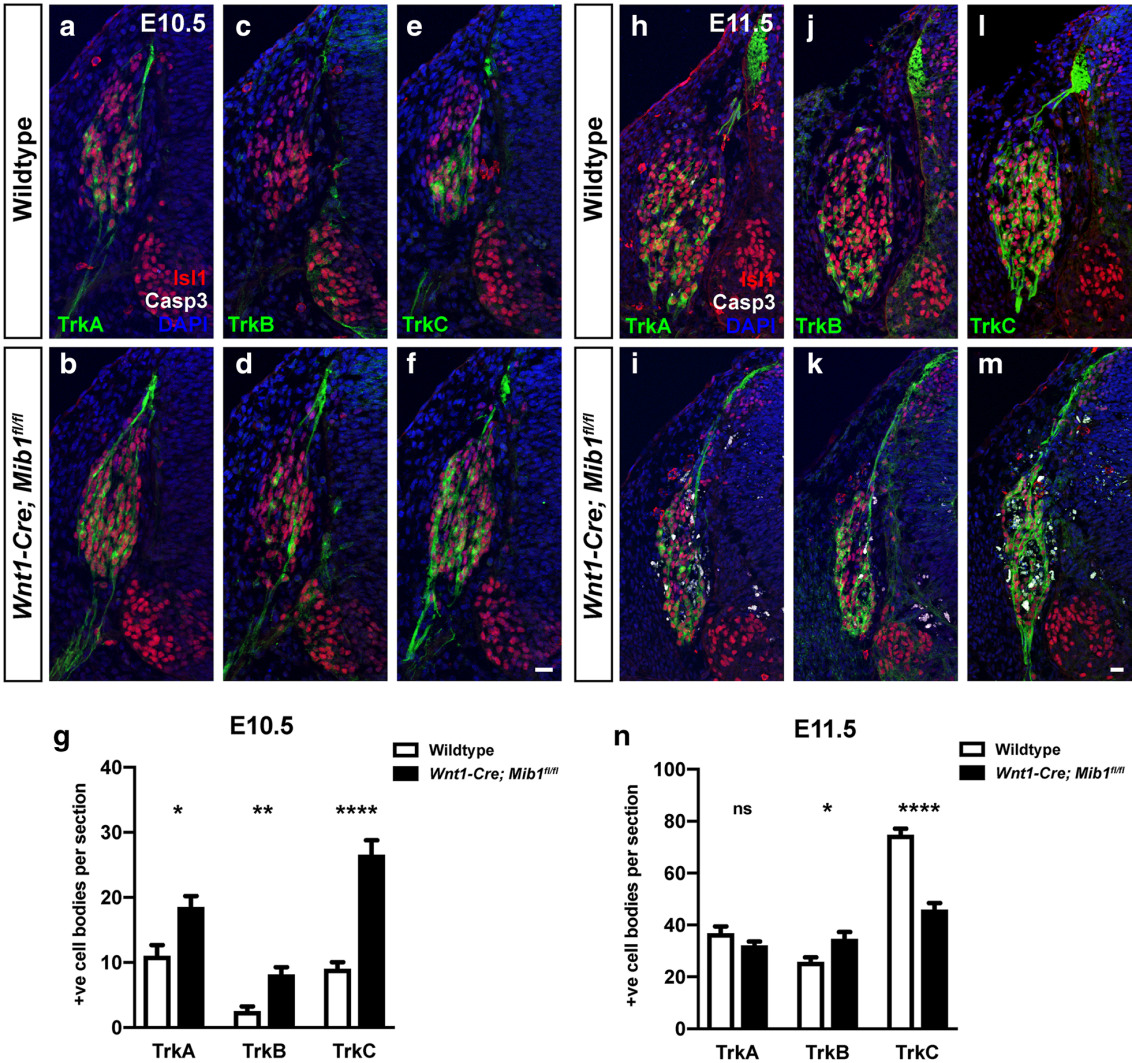
Premature differentiation and aberrant cell death of Trk-positive neuronal subtypes in the DRG of *Wnt1*-*Cre*; *Mib1*^*fl*/*fl*^ embryos (**a**–**f**). Transverse serial sections over the forelimb of wildtype and *Wnt1*-*Cre*; *Mib1*^*fl*/*fl*^ embryos at E10.5 co-immunostained for the neuronal marker Isl1, cell death marker cleaved-Caspase 3 (Casp3), and the neurotrophin receptors TrkA (**a**, **b**), TrkB (**c**, **d**) and TrkC (**e**, **f**). Scale bar = 20 μm. **g** Quantitation of Trk and Isl1-double-positive cell bodies per DRG at E10.5. Number represents mean of n = 3 embryos, with 8 individual sections counted per embryo. *p < 0.05, **p < 0.01, ****p < 0.0001. **h**–**m** Transverse serial sections over the forelimb of wildtype and *Wnt1*-*Cre*; *Mib1*^*fl*/*fl*^ embryos at E11.5 co-immunostained for Isl1, Casp3, and TrkA (**h**, **i**), TrkB (**j**, **k**) and TrkC (**l**, **m**). Scale bar = 20 μm. **n** Quantitation of Trk and Isl1-double-positive cell bodies per DRG at E11.5. Number represents mean of n = 3 embryos, with 8 individual sections counted per embryo, *ns* not significant, *p < 0.05, ****p < 0.0001

Sensory neurons can be divided into nociceptive, mechanoceptive and proprioceptive subtypes based on their expression profile of the neurotrophin (Trk) receptors TrkA, TrkB and TrkC [16]. During DRG morphogenesis, TrkC-positive neurons are the first to be generated, followed temporally by TrkA and TrkB, although there is also a degree of co-expression of Trk receptors within cells at early stages of differentiation [16]. To investigate whether cell death was restricted to a particular sensory neuron subtype, Trk receptor expression was analysed in *Wnt1*-*Cre*; *Mib1*^*fl*/*fl*^ embryos at E10.5 and E11.5. At E10.5, prior to neuronal cell death occurring, expression of all Trk receptors was increased in *Wnt1*-*Cre*; *Mib1*^*fl*/*fl*^ embryos compared to wildtype (n = 3/3; Fig. 3a–g). At E11.5, the dramatic increase in cell death corresponded with a decrease in TrkC-positive neurons in *Wnt1*-*Cre*; *Mib1*^*fl*/*fl*^ embryos, while there was no difference in TrkA numbers, and a slight increase in TrkB-positive neurons remained (n = 3/3; Fig. 3h–n). Taken together, these results suggest initial premature differentiation of neurons occurs upon loss of Mib1, which does not appear to be subtype-specific since expression of all Trk receptors is increased. At E10.5, TrkC showed the most dramatic increase, which is consistent with the TrkC-positive population being the first to be generated. Therefore the selective loss of TrkC-positive neurons at E11.5 may reflect neuronal cell death occurring in a temporal cascade such that the first born TrkC neurons are thus the first to die, leaving the latest born TrkB-positive neurons unaffected at this stage. Alternatively, loss of Mib1 may selectively affect survival of TrkC-positive neurons. Conditional knockout of Mib1 using alternative sensory-specific Cre-drivers may be required to further elucidate a definitive role for Mib1 in neuronal survival and in which particular neuronal subtypes.

### Sox10 is downregulated rapidly during early neural crest cell migration with loss of Mib1

To further investigate the origin of reduced DRG glial progenitors present in *Wnt1*-*Cre*; *Mib1*^*fl*/*fl*^ embryos at E10.5, we investigated early stages of trunk neural crest cell development from E9.25 (20 somite) to E9.75 (26 somite). As well as a marker of glial cells, Sox10 is expressed in neural crest cells immediately upon their delamination from the neural tube, and is hence widely used as a neural crest cell marker. This has made study of neural crest-derived glial progenitors somewhat challenging as the Sox10 marker is unable to distinguish multi-potent neural crest progenitor cells from specified glia. An alternative widely used neural crest cell marker is activating enhancer binding protein 2 alpha (AP2α) [17], which like Sox10, is expressed as neural crest cells delaminate from the neural tube and during their early migration toward the forming DRG.

**Fig. 4:**
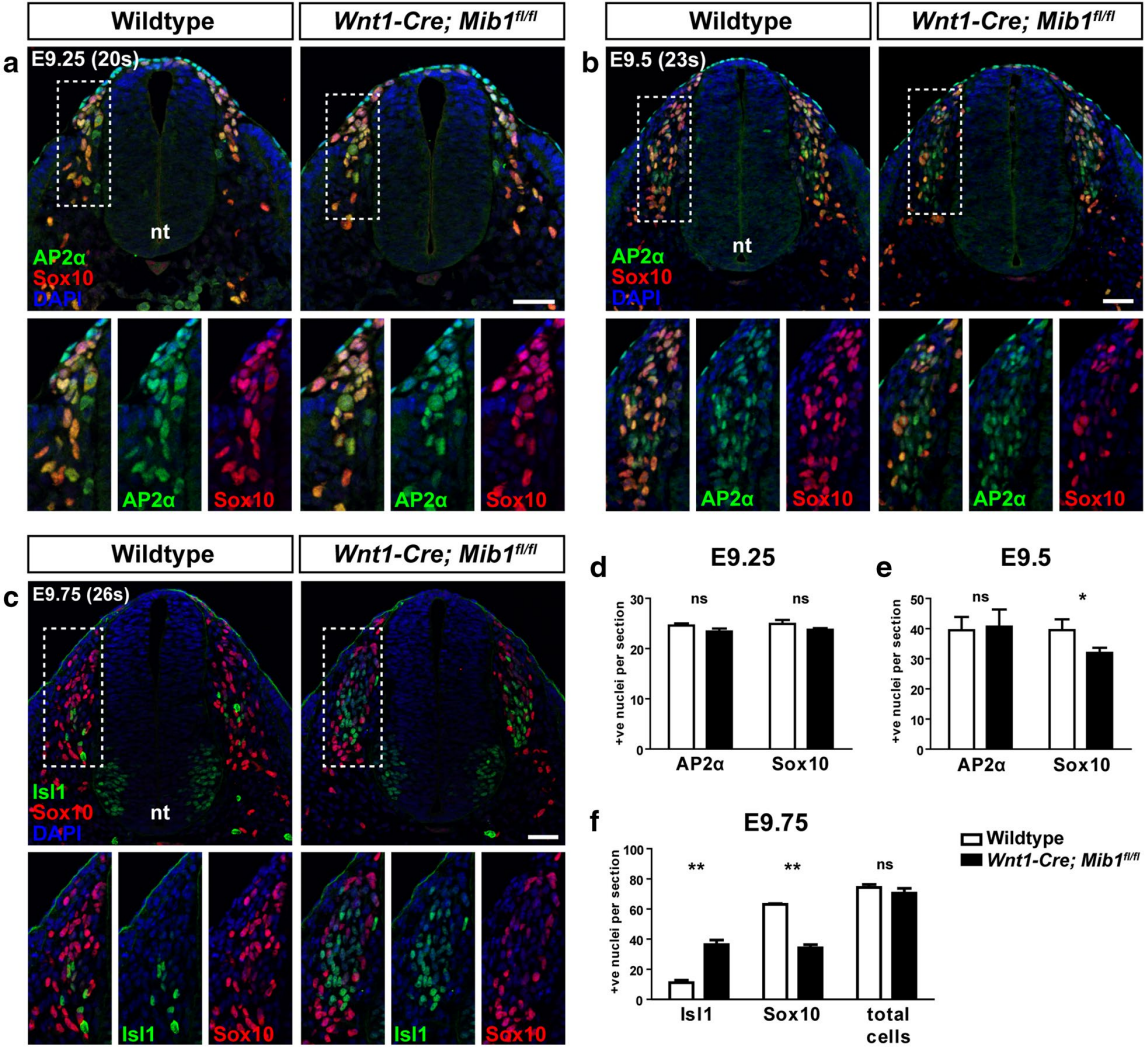
Sox10 is rapidly downregulated in *Wnt1*-*Cre*; *Mib1*^*fl*/*fl*^ embryos during early neural crest cell migration. **a** Transverse sections over the forelimb region of wildtype and *Wnt1*-*Cre*; *Mib1*^*fl*/*fl*^ embryos at E9.25 (20 somite pairs) co-immunostained for the migrating neural crest cell markers AP2α and Sox10. Inset images in lower panels show higher magnification of the boxed region in upper panels. Scale bar = 50 μm. **b** Transverse sections of embryos at E9.5 (23 somite pairs) co-immunostained for AP2α and Sox10. Scale bar = 50 μm. **c** Transverse sections at E9.75 (26 somite pairs) co-immunostained for Isl1 and Sox10. Scale bar = 50 μm. **d** Quantitation of AP2α-positive and Sox10-positive cells per migrating neural crest stream at E9.25. Number represents mean of n = 3 embryos, with over 20 individual sections counted per embryo. ns = not significant. **e** Quantitation of E9.5 embryos, n = 4 embryos. *p < 0.05. **f** Quantitation of E9.75 embryos, n = 3 embryos, *nt* neural tube, **p < 0.01

Analysis of AP2α and Sox10-positive neural crest cells at E9.25 revealed no deficiency of early neural crest cell delamination and migration in the trunk of *Wnt1*-*Cre*; *Mib1*^*fl*/*fl*^ embryos (n = 3/3; Fig. 4a, d). Likewise, at E9.5, there was no difference in the number of AP2α-positive neural crest cells between wildtype and *Wnt1*-*Cre*; *Mib1*^*fl*/*fl*^ embryos (n = 4/4; Fig. 4b, e). However, there was a notable decrease in the number of Sox10-positive neural crest cells in *Wnt1*-*Cre*; *Mib1*^*fl*/*fl*^ embryos at this stage (Fig. 4b, e), suggesting a subset of AP2α-positive neural crest cells had begun to downregulate expression of Sox10 during migration between E9.25 and E9.5. To determine the fate of these AP2α-positive, Sox10-negative neural crest cells, trunk neural crest cell development was analysed slightly later at E9.75. While in wildtype embryos a small proportion of cells had begun to express the neuronal marker Isl1 with the majority of cells still expressing Sox10, in *Wnt1*-*Cre*; *Mib1*^*fl*/*fl*^ embryos around half of all neural crest cells populating the DRG had begun to express Isl1, at the expense of Sox10 (n = 3/3; Fig. 4c, f). This is consistent with the premature expression of Trk receptors observed at E10.5. Taken together, this data suggests that initial neural crest cell specification, delamination and migration occurs normally in *Wnt1*-*Cre*; *Mib1*^*fl*/*fl*^ embryos, however, shortly upon exiting the neural tube, a subset of neural crest cells rapidly lose expression of Sox10, and as a consequence differentiate into neurons, without a change in the overall number of neural crest progenitors populating the DRG. This also implies that neuroglial fate choice of neural crest cells forming the DRG may occur earlier in development than previously reported [4, 8, 18].

### Neural crest cells biased toward the sensory lineage form correctly in the absence of Mib1

While our previous results suggest that neural crest cell formation and delamination is not affected in the absence of Mib1 they did not address a potential role for Notch signalling in regulating the bias of neural crest cells toward the sensory versus sympathetic lineage. The pro-neural transcription factor *Neurogenin 2* (*Ngn2*) is required to specify neural crest cells toward the sensory lineage and is expressed in neural crest cells as they delaminate and migrate toward the DRG [2, 19]. A similar number of neural crest cells were found to express *Ngn2* in *Wnt1*-*Cre*; *Mib1*^*fl*/*fl*^ and control embryos at E9.5 (23 somites) and E10.5 (n = 3/3; Additional file 1: Fig. S2). Mib1 is therefore dispensible for regulating expression of *Ngn2* and biasing neural crest cells toward the sensory lineage.

### Active Notch signalling and neuroglial fate choice occurs immediately as neural crest cells exit the neural tube

Delta-like 1 (DLL1) has previously been characterised as the major Notch ligand directing neuroglial fate choice during DRG development [8]. Mib1 functions to regulate endocytosis of Notch ligands upon signalling to Notch receptors on adjacent cells, and this endocytosis is essential to enable correct Notch signalling in the receiving cell. Absence of Mib1 has previously been shown to result in accumulation of Notch ligands at the cell surface [12], and hence this protein accumulation is reflective of cells that are attempting to actively signal to adjacent cells.

**Fig. 5:**
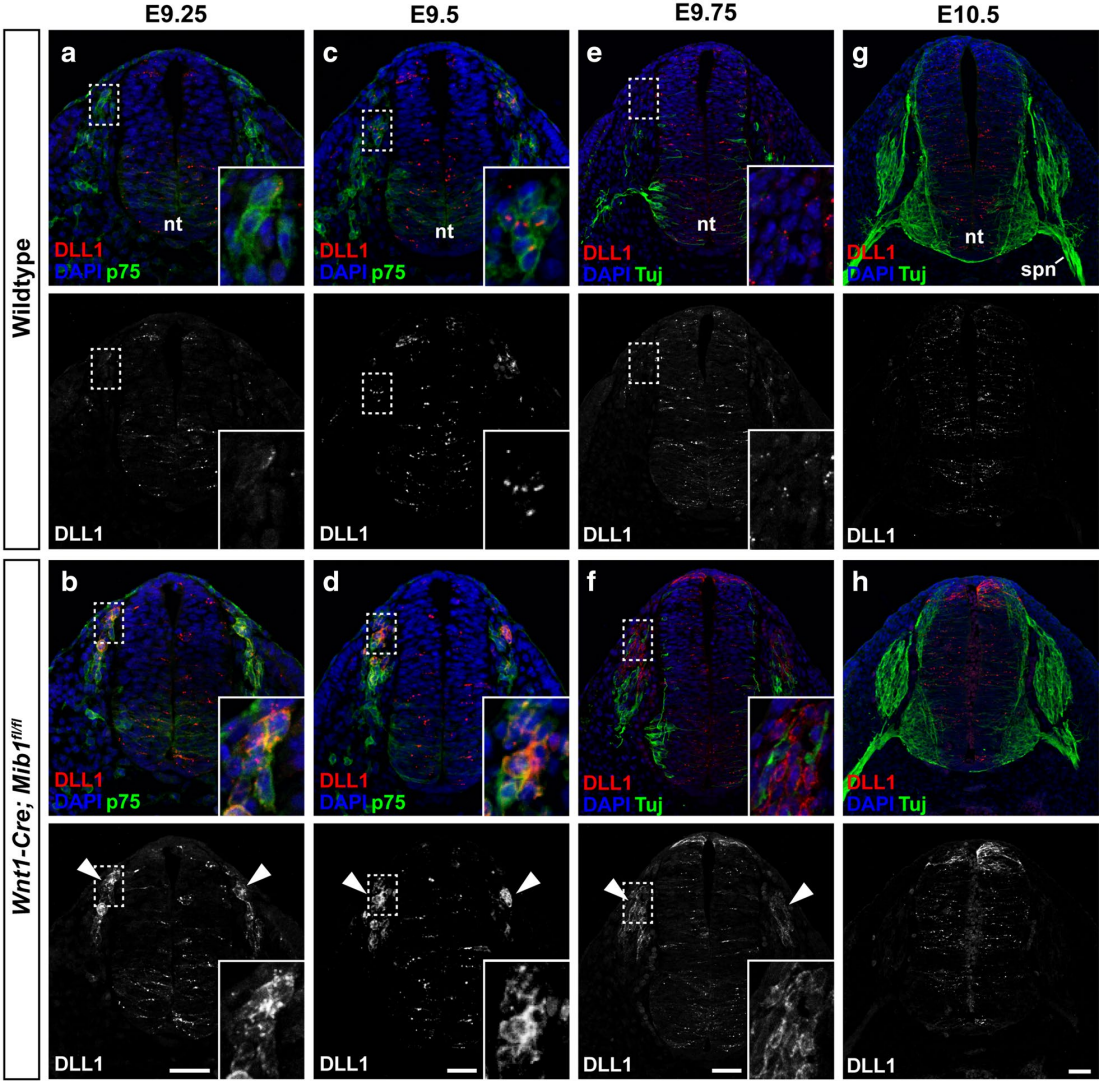
DLL1 accumulation in *Wnt1*-*Cre*; *Mib1*^*fl*/*fl*^ embryos reveals timing of DLL1-Notch signalling during early neural crest cell migration. **a** Transverse sections through the forelimb region of wildtype embryos at E9.25 (20 somite pairs) co-immunostained for DLL1 and the neural crest cell marker p75. Lower panel shows DLL1 staining alone for clarity. Inset image is a higher magnification of the boxed region. **b** Sections of *Wnt1*-*Cre*; *Mib1*^*fl*/*fl*^ embryos at E9.25 co-immunostained for DLL1 and p75. Arrowheads indicate regions with increased DLL1 accumulation compared to wildtype. **c**, **d** Wildtype and *Wnt1*-*Cre*; *Mib1*^*fl*/*fl*^ embryos at E9.5 (23 somites) co-immunostained for DLL1 and p75. **e**, **f** Wildtype and *Wnt1*-*Cre*; *Mib1*^*fl*/*fl*^ embryos at E9.75 (26 somites) co-immunostained for DLL1 and the axonal/neuronal marker Tuj. **g**, **h** Wildtype and *Wnt1*-*Cre*; *Mib1*^*fl*/*fl*^ embryos at E10.5 co-immunostained for DLL1 and Tuj, n = 3 embryos/genotype. *nt* neural tube; *spn* spinal nerve. All scale bars = 50 μm

Analysis of DLL1 expression during early neural crest cell development, through to stages of DRG condensation, revealed dynamic changes in DLL1 localisation in *Wnt1*-*Cre*; *Mib1*^*fl*/*fl*^ embryos when compared to wildtype (Fig. 5). At E9.25 (20 somites), co-immunostaining with the neural crest marker p75 neurotrophin receptor (p75) and DLL1 revealed a salt-and-pepper pattern of DLL1 expression within the migrating streams of trunk neural crest cells of wildtype embryos (Fig. 5a). However, in *Wnt1*-*Cre*; *Mib1*^*fl*/*fl*^ embryos, DLL1 was highly expressed in most migrating neural crest cells, and was particularly abundant in the dorsal-most cells that had just delaminated from the neural tube (n = 3/3; arrowheads, Fig. 5b). This expression pattern was maintained in wildtype and *Wnt1*-*Cre*; *Mib1*^*fl*/*fl*^ embryos at E9.5 (n = 3/3; 23 somites) (Fig. 5c, d). This suggests active DLL1-Notch signalling is occurring between multipotent neural crest progenitors at the earliest stages of neural crest migration post-delamination.

At later stages of neural crest migration and early DRG condensation, co-immunostaining for Tuj1 and DLL1 revealed minor changes in DLL1 localisation and abundance in the DRG compared to earlier stages of development. At E9.75 (26 somites), premature differentiation of neuronal cells in the ventral-most region of the forming DRG was evident in *Wnt1*-*Cre*; *Mib1*^*fl*/*fl*^ embryos as shown by increased Tuj1 immunostaining compared to wildtype (n = 3/3; Fig. 5e, f). At this stage of development, there was also increased DLL1 immunostaining in the dorsal-most neural crest progenitors populating the DRG in *Wnt1*-*Cre*; *Mib1*^*fl*/*fl*^ embryos (n = 3/3; solid arrowheads, Fig. 5f). By E10.5, minimal DLL1 immunostaining was evident in the DRG in both wildtype and *Wnt1*-*Cre*; *Mib1*^*fl*/*fl*^ embryos (n = 3/3; Fig. 5g, h), suggesting cells in the DRG are not actively undergoing DLL1-Notch signalling at this stage of development. This further suggests DLL1-Notch signalling to determine DRG cell neuroglial fate choice is executed at earlier stages of development, primarily during neural crest cell delamination and migration between E9.25 and E9.5.

The accumulation of DLL1 in the neural crest cells of *Wnt1*-*Cre*; *Mib1*^*fl*/*fl*^ embryos is also due to a specific effect of Mib1 on the DLL1 ligand, as other ligands such as Jagged 1 (Jag1) did not show aberrant localisation or accumulation in *Wnt1*-*Cre*; *Mib1*^*fl*/*fl*^ embryos (n = 3/3; Additional file 1: Fig. S3). An accumulation of DLL1 was also observed at the dorsal ridge of the neural tube in *Wnt1*-*Cre*; *Mib1*^*fl*/*fl*^ embryos at E9.75–E10.5 (n = 3/3; Fig. 5f, h), which may suggest a role for Notch signalling in roof plate formation, however this likely occurs independently of DRG development.

**Fig. 6:**
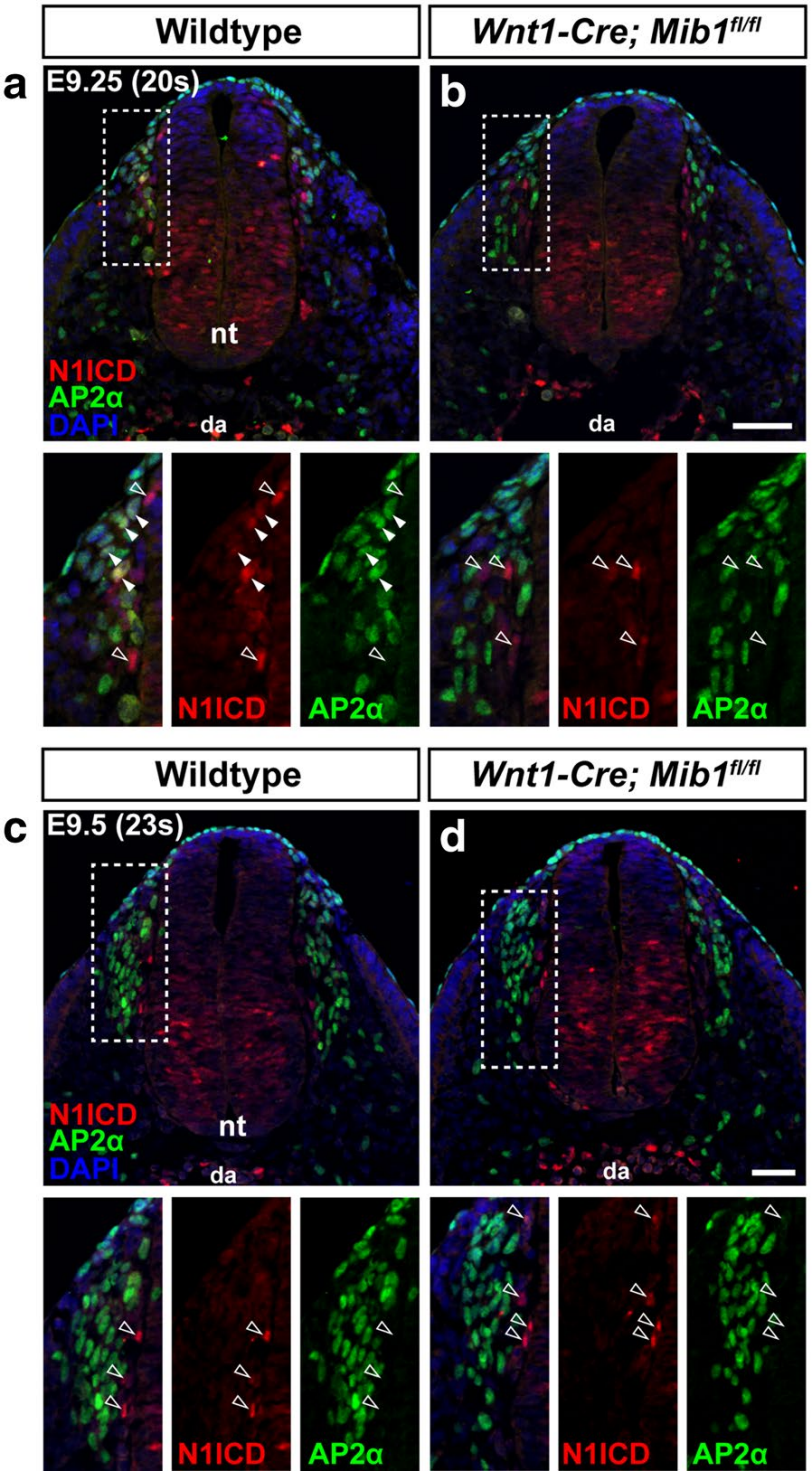
Notch signalling is active in neural crest cells at the earliest stages of migration. **a** Transverse sections through the forelimb region of wildtype embryos at E9.25 (20 somite pairs) co-immunostained for N1ICD and the neural crest cell marker AP2α. Inset images in lower panels show higher magnification of the boxed region in upper panel. Solid arrowheads highlight neural crest cells undergoing active Notch signalling (AP2α and N1ICD positive). Open arrowheads highlight non-neural crest cell types within the migrating neural crest stream that are undergoing active Notch signalling (AP2α-negative, N1ICD-positive). nt, neural tube; da, dorsal aorta. **b** Sections of *Wnt1*-*Cre*; *Mib1*^*fl*/*fl*^ embryos at E9.25 co-immunostained and labelled for N1ICD and AP2α. Scale bar = 50 μm. **c**, **d** Sections of wildtype and *Wnt1*-*Cre*; *Mib1*^*fl*/*fl*^ embryos at E9.5 (23 somite pairs) co-immunostained for N1ICD and AP2α. n = 3 embryos/genotype. Scale bar = 50 μm

Upon stimulation with ligand, the Notch receptor is cleaved by γ-secretase to generate an intracellular fragment known as the Notch Intracellular Domain (NICD) which translocates to the nucleus and associates with other protein partners, such as RBPJ and modulation of Notch signalling by mastermind-like (MAML), to activate target gene transcription. To investigate active Notch signalling during early trunk neural crest cell development, sections were co-immunostained for Notch1 ICD (N1ICD) and AP2α. In wildtype embryos at E9.25, many of the early migrating (dorsal-most) AP2α-positive neural crest cells were positive for N1ICD (solid arrowheads, Fig. 6a), indicating these cells are undergoing active Notch signalling and have been stimulated by ligand. In *Wnt1*-*Cre*; *Mib1*^*fl*/*fl*^ embryos however, none of the AP2α-positive neural crest cells were positive for N1ICD, indicating that in the absence of active ligands, Notch signalling is not induced (n = 3/3; Fig. 6b). This further suggests that the accumulated DLL1 protein present at this stage of development in *Wnt1*-*Cre*; *Mib1*^*fl*/*fl*^ embryos is non-functional. As expected, Notch signalling is not abrogated in non-neural crest derivatives in *Wnt1*-*Cre*; *Mib1*^*fl*/*fl*^ embryos, as evidenced by positive N1ICD staining in the neural tube, endothelial cells of the dorsal aorta (da), and other AP2α-negative cell types present within the migrating neural crest stream (open arrowheads, Fig. 6a, b). Notch signalling was also analysed at E9.5, however none of the neural crest cells in wildtype or *Wnt1*-*Cre*; *Mib1*^*fl*/*fl*^ embryos were positive for N1ICD (n = 3/3; Fig. 6c, d), suggesting this important DLL1-Notch1 signalling event occurs immediately after neural crest cells have delaminated from the neural tube. Expression of the Notch1 receptor remained unchanged in *Wnt1*-*Cre*; *Mib1*^*fl*/*fl*^ embryos (Additional file 1: Fig. S4), further suggesting the changes in N1ICD observed were a direct result of inactive ligand-receptor signalling.

## Discussion

In this study, we show that removal of *Mib1*, and hence Notch ligand activity, in neural crest-derived tissue causes profound DRG defects. Our findings suggest that a loss of Sox10 expression in a subset of early migrating neural crest cells leads to a reduction of glial cells in the DRG, which is accompanied by neuronal cell death, resulting in severe DRG hypoplasia in later development. While Notch signalling has previously been demonstrated to play an important role in DRG and in particular glial development [5, 6], our study brings into question the timing of this neuroglial fate choice. By taking advantage of DLL1 ligand accumulation in the *Mib1* knockout model, and by demonstrating the timing of N1ICD presence in the nucleus of neural crest cells, our data suggests that active Notch signalling promotes glial cell fate decisions much earlier in DRG development than originally anticipated.

Previous models of neuroglial fate choice in the DRG are largely based on the premise that neural crest cells migrate from the neural tube and coalesce into a condensed DRG structure as an unspecified population of progenitors [3, 4]. Under this model, most nascent neural crest cells are predicted to adopt a neuronal or glial fate after this condensed structure has formed. Another common point of view is that neuronal differentiation occurs before glial specification, where neuronally biased DLL1 expressing cells residing in the core of the DRG signal via Notch to unspecified cells in the DRG periphery to prevent their neuronal differentiation and promote glial specification [3, 8, 20, 21]. A number of our findings instead fit with the notion that Notch signalling occurs between neural crest cells at the earliest stages of neural crest migration prior to coalescing into the nascent DRG structure and before establishment of the core versus periphery. Thus, Notch activity was readily detected in neural crest cells that had recently delaminated at E9.25 but not in the neural crest cells coalescing within the region of the DRG at any stage examined. Following the absence of Notch signalling in *Wnt1*-*Cre*; *Mib1*^*fl*/*fl*^ embryos at E9.25 we also identified an increase in Isl1 positive neurons in the dorsal regions of the DRG at E9.75. The observation that these ectopic Isl1 positive cells colocalise with Dll1 positive neural crest cells that have attempted to activate Notch during their migration further suggests that these cells aberrantly form neurons. However, in the absence of lineage tracing cells that have activated Notch during early neural crest cell migration it remains possible that the neuronal or glial fate choice could also be influenced at earlier or later stages of development. Indeed, a limitation of our mouse model is that Mib1 has only been removed in neural crest cells as they delaminate which has not allowed us to address any potential roles for Notch activity in premigratory neural crest in the dorsal neural tube at earlier stages of development.

The question of whether neural crest progenitors are fate-restricted even before their exit from the neural tube is a heavily debated topic. Spatial transcriptional profiling has revealed distinct sub-populations of neural crest stem cells in the dorsal neural tube, however their concomitant expression of both pluripotency and lineage markers suggests these populations are open to many fate-choices [22]. Indeed, both historical and more recent genetic lineage tracing studies have concluded that the majority of individual premigratory and migratory neural crest cells are multipotent in vivo [23, 24]. However, other studies suggest that the position within and timing of delamination from the neural tube determines the fate of pre-migratory neural crest cells [25], which is also supported by the findings that Ngn2 expressing cells within the dorsal neural tube are biased toward the sensory lineage [2]. In our present study, N1ICD and Dll1 were not detected in the premigratory neural crest of the dorsal neural tube. We observe active Notch signalling in neural crest cells only after delamination from the neural tube, which supports the notion that DRG-derivatives are not fate-restricted prior to delamination, and that neuroglial fate choice is determined once neural crest cells are migratory.

Previous work has shown that inhibition of Notch signalling (via deletion of *Rbpj*) in the DRG leads to an increase in the number of neurons at the expense of glial cells [9], implying a change of balance in a binary fate-choice decision. In our model, removal of Notch signalling (via deletion of *Mib1*) lead to a decrease in glial cells, without a concomitant overall increase in neuronal cells at E10.5. We did, however, observe an initial premature differentiation of Isl1-positive neuronal cells at E9.75, and increased numbers of Trk-positive, Isl1-positive co-stained cells at E10.5. Given the number of Isl1-positive neurons remained normal at E10.5, our data suggests the neuronal differentiation/maturation program (Isl1 expression, followed by Trk expression) is executed prematurely in our model. The lack of cell death observed between E9.75 and E10.5 further rules out the possibility that supernumerary neurons are dying. Our data fit with a model in which the number of neurons within the DRG is capped at a fixed number, and that this is reached by E10.5 in *Mib1* conditional knockouts due to premature neuronal differentiation. While the exact nature of this phenomenon remains unclear, the differences between our data and that from *Rbpj* deletion may indicate other roles for Rbpj and/or Mib1 outside of the Notch pathway, or that some Notch activity remains in these models. Notably, Notch has been shown to signal independently of Rbpj in some circumstances [26], and Rbpj has Notch-independent transcriptional targets [27]. Mib1 also has roles outside of regulating Notch ligands [28–30], and it is likely that other ubiquitinated targets of Mib1 are yet to be identified, which may contribute to the phenotype observed.

In addition to inhibiting neuronal differentiation, Notch signalling has also been shown to promote glial cell differentiation in cultured rat neural crest stem cells [7]. Consistent with this idea, we found that Notch activity is necessary to drive expression of the glial markers, Sox10 and FABP7, and to promote gliogenesis. Our data also indicate that one of the primary roles for Notch signalling is to maintain Sox10 expression within a subset of neural crest cells which likely represent glial progenitors. Thus, we show that the number of Sox10 positive cells is reduced prior to an increase in Isl1 positive neuronal precursors. While the mechanisms by which Notch regulates expression of Sox10 remain unknown, it is intriguing that inhibition of Notch in neural crest cells derived from induced pluripotent stem cells leads to Sox10 down regulation [31], and Notch signalling can induce ectopic expression of Sox10 in zebrafish [32]. Taken together with our data this may suggest a direct role for N1ICD in driving Sox10 expression under certain circumstances. Indeed, our own bioinformatic analysis has identified two putative highly conserved RBPj binding sites in intron 1 of the Sox10 locus which may also support this suggestion.

By using Mib1 deletion in this study our intention was to overcome any redundant roles that the Notch ligands may have in neural crest cells. Functional validation of Mib1 deletion with antibodies against two Notch ligands found that Dll1, but not Jag1, is expressed in a sub-set of neural crest cells migrating toward the DRG. This expression pattern is in agreement with previous work implicating a role for Dll1 expressing neural crest cells in myogenesis [33, 34]. Deletion of Dll1 in mice leads to an overlapping phenotype to that identified in our work, including reduced glia and sensory neurons within the DRG [11]. Dll3 also appears to be expressed in a sub-set of neural crest cells within the region of the forming DRG [35], but a role for this ligand in sensory neurogenesis has not specifically been addressed. Whether Dll1 acts as the sole Notch ligand in this fate choice, or if other ligands such as Dll3 are also involved, therefore remains to be explored.

One of the notable defects observed in *Mib1* conditional knockouts in this study was aberrant cell death and depletion of sensory neurons within the DRG after E11.5. A similar temporal progression of neuronal cell death in Sox10 mouse knockouts has been interpreted by some groups to arise as a secondary defect to deficient glial differentiation [36]. However, Sox10 has also been shown to have instructive and pro-survival effects in sensory neurons which may suggest that glia are not required for cell survival during early stages of neuronal differentiation [37, 38]. It is also noteworthy that sensory neuron death within the DRG begins at E11.5 in mouse mutants lacking the pro-survival neurotrophin receptor TrkC independent of abnormalities in glia [16]. As we have removed Mib1 from the precursors of both neurons and glia, and it remains possible that Mib1 may also regulate other proteins outside of the Notch ligands, our data do not allow us to decipher the mechanisms leading to neuronal cell death at this stage.

An interesting observation in our study was mal-positioning of the dorsal root entry zone, misshapen dorsal neural tube, and aberrant axonal tracts in *Wnt1*-*Cre*; *Mib1*^*fl*/*fl*^ embryos (Figs. 2, 3, 5). *Wnt1*-*Cre* drives genetic recombination in the dorsal neural tube prior to neural crest delamination and would be expected to remove Mib1, and therefore Notch ligand activity, in this region. Indeed, accumulation of Dll1 in the dorsal neural tube of *Wnt1*-*Cre*; *Mib1*^*fl*/*fl*^ embryos supports this suggestion. As the N1ICD regulated transcription factor, Hes1, has previously been shown to play an important role in roof plate formation downstream of BMP [39], this may suggest that Notch signalling intersects with BMP signalling to control correct development and patterning of the dorsal neural tube.

## Conclusions

Taken together, our data define the timing at which Notch ligands signal to Notch receptors to determine the neuronal and glial fate of neural crest cells which will give rise to the DRG. This period at which sensory biased neural crest cells choose their fate is much earlier than previously predicted, occurring at the time, or soon after, delamination. Defining a novel cell-autonomous role for the ubiquitin ligase Mib1 in fate specification and formation of the sensory nervous system further highlights the necessity to understand how post translational modifications regulate neural crest cell development [40].

## Methods

### Mouse lines

All experiments on mice were approved by the SA Pathology and University of South Australia Animal Ethics Committee (Research Ethics License 51-15). Mice were kept in open top conventional cages, on a 12-h light/dark cycle, with free access to food and water. To obtain embryos of defined gestational ages, mice were mated in the evening, and the morning of vaginal plug formation was counted as embryonic day (E) 0.5. Pregnant dams were humanely euthanized at relevant days post vaginal plug detection by CO2 inhalation and cervical dislocation. To lineage trace neural crest cells and their derivatives, we crossed *Wnt1*-*Cre* mice [41] to *Z*/*EG* mice [42]. To delete *Mib1* specifically in neural crest cells, we mated *Mib1*^*fl*/+^ males carrying a heterozygous *Wnt1*-*Cre* transgene to *Mib1*^*fl*/*fl*^ female mice [43]. At least 3 embryos/age/genotype were analysed for each experiment.

### Immunohistochemistry

Embryos were fixed in 4% paraformaldehyde for 2 h or overnight at 4 °C, cryopreserved in 20% sucrose, and embedded in OCT compound for cryosectioning. Sections were blocked in 10% Dako serum-free blocking reagent or 10% goat serum in PBS 0.1% TritonX-100 (with some exceptions, see below), followed by incubation in primary antibody for 2 h at room temperature or overnight at 4 °C. Fluorescent Alexafluor-conjugated secondary antibodies were incubated for 1 h at room temperature. Sections were mounted in Prolong Diamond antifade with 4′,6-diamidino-2-phenylindole (DAPI). The following primary antibodies were used: Chicken anti-EGFP, 1:1000 (Abcam ab13970); mouse anti-Tuj1, 1:750 (Sigma-Aldrich T5076); goat anti-Sox10, 1:100 (Santa Cruz sc-17342); goat anti-Sox10, 1:100 (R&D Systems AF2864); mouse anti-Isl1, 1:50 (DSHB 40.3A4); rabbit anti-cleaved Caspase-3, 1:500 (Cell Signaling Technologies 9661); rabbit anti-FABP7, 1:200 (Cell Signaling Technologies 13347); goat anti-TrkA, 1:50 (R&D Systems AF1056); goat anti-TrkB, 1:200 (R&D Systems AF1494); goat anti-TrkC, 1:200 (R&D Systems AF1404); mouse anti-AP2α, 1:20, goat serum block (DSHB 3B5); sheep anti-DLL1, 1:200 (R&D Systems AF5026); rabbit anti-p75, 1:250 (Abcam 52987); goat anti-Jag1, 1:200 (R&D Systems AF599); rabbit anti-N1ICD, 1:100 (Cell Signaling Technologies 4147); sheep anti-Notch1, 1:200 (R&D Systems AF5267).

### Antigen retrieval

Immunostaining for AP2α and N1ICD required antigen retrieval [44]. Cryosections were incubated in 10 mM Sodium Citrate pH6.0 at 90 °C for 20 min, and then cooled to room temperature before proceeding with immunohistochemistry procedure as above.

### Tyramide amplification

Immunostaining for N1ICD required tyramide signal amplification, performed using Invitrogen Alexa Fluor 555 Tyramide SuperBoost Kit (B40923). Following primary antibody incubation, sections were incubated with goat anti-rabbit horse radish peroxidase (HRP), then tyramide signal amplification performed for 30 min, following manufacturer’s instructions.

### TUNEL staining

Following immunohistochemical staining, sections were stained using Roche In Situ Cell Death Detection Kit TMR red following manufacturer’s instructions for 1 h at 37 °C.

### In situ hybridisation

Section in situ hybridisation was performed as described [45]. Riboprobes were transcribed from plasmids containing the cDNA sequence for *Ngn2* [46].

### Quantitation of cell numbers

Transverse sections spanning the anterior to posterior limits of 2–3 DRG were collected over the forelimbs of E9.25-11.5 embryos. The total number of Sox10 positive, Isl1 positive, TUNEL positive, Casp3 positive, Trk positive or AP2α positive cells were manually counted from 20× images from each section spanning the limits of each DRG. Approximately 15–20 sections each from at least 3 embryos per age/genotype were analysed. Data are represented as the number of antigen-positive cells per 12 μm section per DRG. All data are presented as mean ± SEM and analysed using Student’s *t* test. In all studies a *p* value of < 0.05 was considered to be statistically significant.

## Additional file

**Additional file 1: Fig**. **S1**. Glial cell maturation is inhibited in the DRG of *Wnt1*-*Cre*; *Mib1*^*fl*/*fl*^ mice. (**A**) Transverse sections of wildtype and *Wnt1*-*Cre*; *Mib1*^*fl*/*fl*^ embryos at E11.5 co-immunostained for the axonal marker Tuj1 and the glial marker FABP7. (**B**) FABP7 staining shown alone for clarity. Scale bar = 50 μm. (**C**) Transverse sections of wildtype and *Wnt1*-*Cre*; *Mib1*^*fl*/*fl*^ embryos at E13.5 co-immunostained for Tuj1 and FABP7. (**D**) FABP7 staining shown alone for clarity. n = 3 embryos/genotype. nt, neural tube; spn, spinal nerve. Scale bar = 100 μm. **Fig**. **S2**. Neural crest specification toward the sensory lineage in conserved in *Wnt1*-*Cre*; *Mib1*^*fl*/*fl*^ mice. In situ hybridisation for *Ngn2* on transverse sections of wildtype and *Wnt1*-*Cre*; *Mib1*^*fl*/*fl*^ embryos at E9.5 and E10.5. n = 3 embryos/genotype. nt, neural tube. Scale bar = 50 μm. **Fig**. **S3**. **The Notch ligand Jag1 remains unchanged in neural crest upon loss of Mib1**. Transverse sections of wildtype and *Wnt1*-*Cre*; *Mib1*^*fl*/*fl*^ embryos at E9.25 co-immunostained for the neural crest cell marker p75 and Jag1. n = 3 embryos/genotype. nt, neural tube. Scale bar = 50 μm. **Fig**. **S4**. **Total Notch1 receptor levels remain unchanged in neural crest upon loss of Mib1**. Transverse sections of wildtype and *Wnt1*-*Cre*; *Mib1*^*fl*/*fl*^ embryos at E9.5 co-immunostained for the neural crest cell marker p75 and Notch1. n = 3 embryos/genotype. nt, neural tube. Scale bar = 50 μm.

## Abbreviations

DRG: dorsal root ganglia
Mib1: Mindbomb 1
DLL1: Delta-like 1
RBPJ: recombination signal binding protein for immunoglobulin kappa J region
Sox10: SRY-related HMG-box 10
N1ICD: Notch1 intracellular domain
E: embryonic day
EGFP: enhanced green fluorescent protein
Tuj1: neuron specific class-III b tubulin
Isl1: islet 1
TUNEL: terminal deoxynucleotidyl transferase dUTP nick end labelling
Casp3: cleaved caspase 3
AP2a: activating enhancer binding protein 2 alpha
Ngn2: Neurogenin 2
p75: p75 neurotrophin receptor
Jag1: Jagged 1
MAML: modulation of Notch signalling by mastermind-like
da: dorsal aorta
FABP7: fatty acid binding protein 7
PBS: phosphate buffered saline
DAPI: 4′,6-diamidino-2-phenylindole
HRP: horse radish peroxidase
