# Supplementary figures and images for: Notch signalling defines dorsal root ganglia neuroglial fate choice during early neural crest cell migration
Source: BMC Neurosci. 2019 Apr 29;20:21. doi: 10.1186/s12868-019-0501-0 (PMC6489353; doi:10.1186/s12868-019-0501-0)

Fig. S1

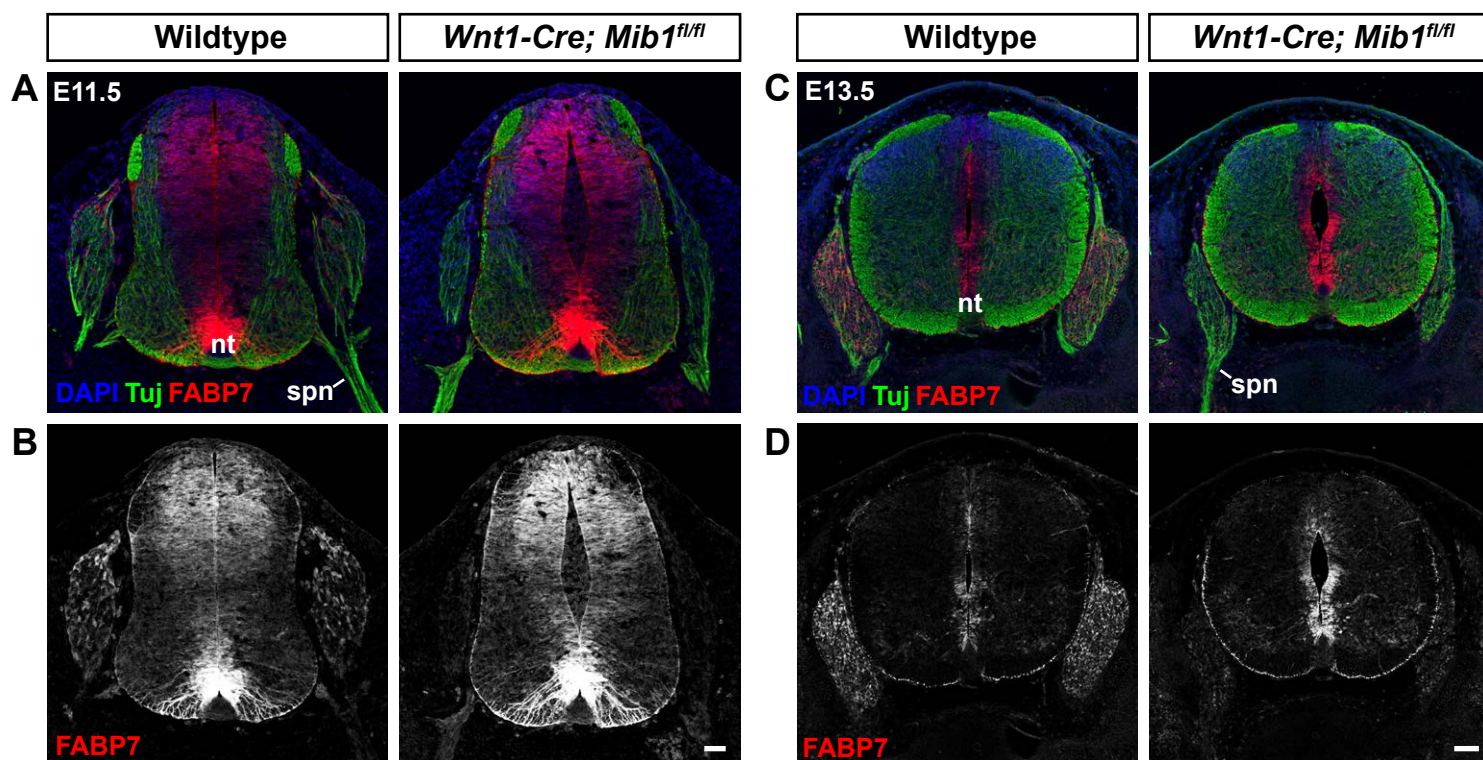

**Fig. S2**

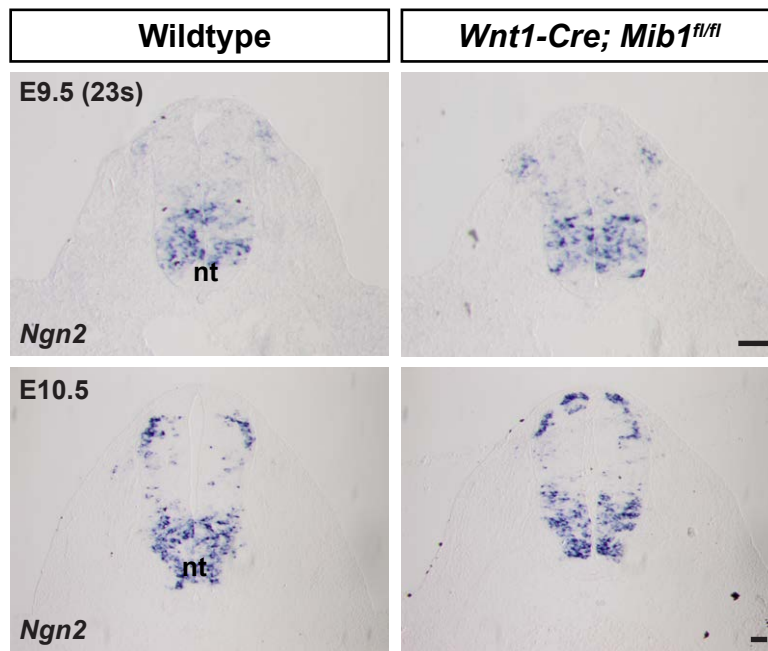

Fig. S3

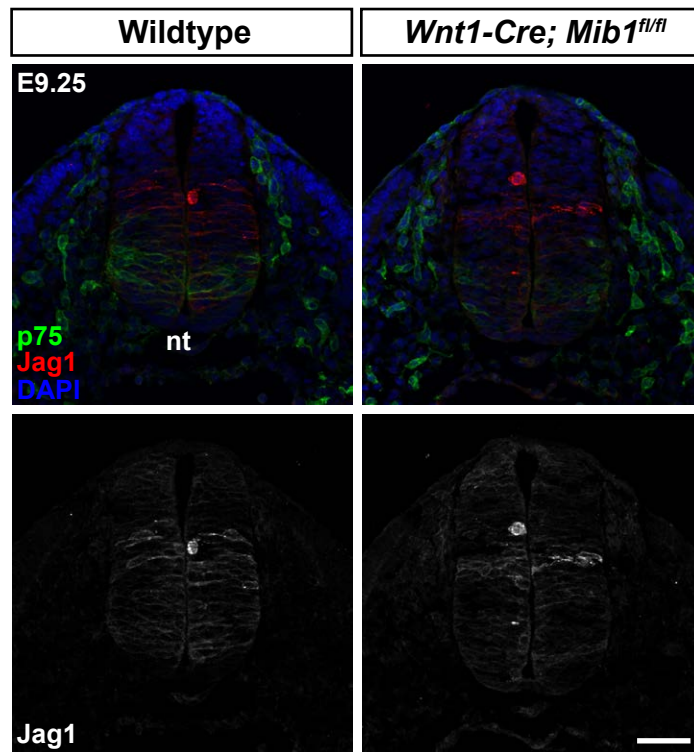

**Fig. S4**

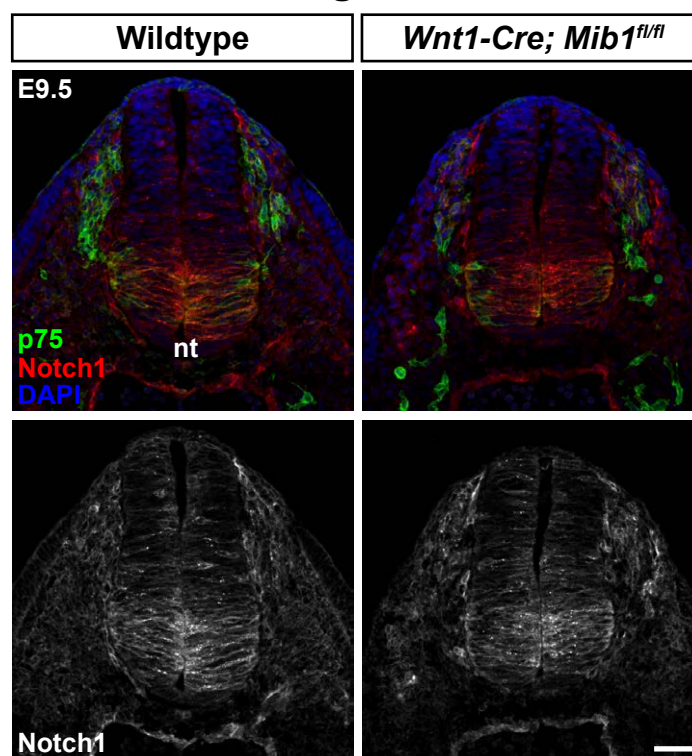

Supplement: Supplementary file 1 — Additional file 1: Fig. S1. Glial cell maturation is inhibited in the DRG of Wnt1-Cre; Mib1fl/fl mice. (A) Transverse sections of wildtype and Wnt1-Cre; Mib1fl/fl embryos at E11.5 co-immunostained for the axonal marker Tuj1 and the glial marker FABP7. (B) FABP7 staining shown alone for clarity. Scale bar = 50 μm. (C) Transverse sections of wildtype and Wnt1-Cre; Mib1fl/fl embryos at E13.5 co-immunostained for Tuj1 and FABP7. (D) FABP7 staining shown alone for clarity. n = 3 embryos/genotype. nt, neural tube; spn, spinal nerve. Scale bar = 100 μm. Fig. S2. Neural crest specification toward the sensory lineage in conserved in Wnt1-Cre; Mib1fl/fl mice. In situ hybridisation for Ngn2 on transverse sections of wildtype and Wnt1-Cre; Mib1fl/fl embryos at E9.5 and E10.5. n = 3 embryos/genotype. nt, neural tube. Scale bar = 50 μm. Fig. S3. The Notch ligand Jag1 remains unchanged in neural crest upon loss of Mib1. Transverse sections of wildtype and Wnt1-Cre; Mib1fl/fl embryos at E9.25 co-immunostained for the neural crest cell marker p75 and Jag1. n = 3 embryos/genotype. nt, neural tube. Scale bar = 50 μm. Fig. S4. Total Notch1 receptor levels remain unchanged in neural crest upon loss of Mib1. Transverse sections of wildtype and Wnt1-Cre; Mib1fl/fl embryos at E9.5 co-immunostained for the neural crest cell marker p75 and Notch1. n = 3 embryos/genotype. nt, neural tube. Scale bar = 50 μm. [file 12868_2019_501_MOESM1_ESM.pdf]
